# Verification of Non-Invasive Blood Glucose Measurement Method Based on Pulse Wave Signal Detected by FBG Sensor System

**DOI:** 10.3390/s17122702

**Published:** 2017-11-23

**Authors:** Shintaro Kurasawa, Shouhei Koyama, Hiroaki Ishizawa, Keisaku Fujimoto, Shun Chino

**Affiliations:** 1 Graduate School of Science and Technology, Shinshu University, 3-15-1 Tokida, Ueda, Nagano 386-8567, Japan; 17st205a@shinshu-u.ac.jp (S.K.); 17st108j@shinshu-u.ac.jp (S.C.); 2 Faculty of Textile Science and Technology, Shinshu University, 3-15-1 Tokida, Ueda, Nagano 386-8567, Japan; 3 Institute for Fiber Engineering, Shinshu University, 3-15-1 Tokida, Ueda, Nagano 386-8567, Japan; zawa@shinshu-u.ac.jp; 4 Department of Clinical Laboratory Sciences, School of Health Sciences, Shinshu University, 3-1-1 Asahi, Matsumoto, Nagano 390-8621, Japan; keisaku@shinshu-u.ac.jp

**Keywords:** fiber Bragg grating, pulse wave signal, blood glucose level, non-invasive measurement, partial least squares regression, blood flow

## Abstract

This paper describes and verifies a non-invasive blood glucose measurement method using a fiber Bragg grating (FBG) sensor system. The FBG sensor is installed on the radial artery, and the strain (pulse wave) that is propagated from the heartbeat is measured. The measured pulse wave signal was used as a collection of feature vectors for multivariate analysis aiming to determine the blood glucose level. The time axis of the pulse wave signal was normalized by two signal processing methods: the shortest-time-cut process and 1-s-normalization process. The measurement accuracy of the calculated blood glucose level was compared with the accuracy of these signal processing methods. It was impossible to calculate a blood glucose level exceeding 200 mg/dL in the calibration curve that was constructed by the shortest-time-cut process. In the 1-s-normalization process, the measurement accuracy of the blood glucose level was improved, and a blood glucose level exceeding 200 mg/dL could be calculated. By verifying the loading vector of each calibration curve to calculate the blood glucose level with a high measurement accuracy, we found the gradient of the peak of the pulse wave at the acceleration plethysmogram greatly affected.

## 1. Introduction

In recent years, the number of diabetic patients has been steadily increasing worldwide. This increase has led to strong needs for a rapid, painless, risk-free self-blood-glucose measurement method [1,2,3]. Research and development for measurement methods based on spectroscopy began in the 1970s. It is well known that attenuated total reflectance [4] or near-infrared diffuse reflectance spectroscopy [5] had been applied to realize non-invasive blood glucose monitoring, mainly for diabetic patients. Since then, various developments have been made. A microwave measurement system has been proposed to monitor blood glucose non-invasively. Microwave sensor technologies were studied based on the frequency dependence of amplitude with the subject’s thumb being placed at a fixed point on an open-terminated spiral-shaped micro strip line [6,7]. In the analysis method of calculating the blood glucose level, a method of calculating blood glucose level with high accuracy by applying artificial neural network (ANN) [8], partial least squares regression (PLSR) [9], and the like has been studied. In the measurement method using light, a method of calculating blood glucose level by Raman spectroscopy has been reported [10,11]. In report of Spegazzini, Raman spectra were recorded at regular 5 min intervals from the forearms of these volunteers, blood glucose concentrations were calculated by using the improved concentration independent calibration (iCONIC) approach with Raman spectra [10]. However, since these are methods of irradiating light on the body, there is a danger that the measurement accuracy of the blood glucose level will be influenced by the surface condition and body temperature of the skin of the subject. On the other hand, a measured method for blood glucose with strain of pulse wave has not yet been reported.

The paper proposes a new non-invasive blood glucose measurement method, which is based on a pulse wave signal detected using a fiber Bragg grating (FBG) sensor, which is a highly sensitive strain sensor. A pulse wave is a measurement of a pressure change or a volume change of a peripheral blood vessel propagated by a heartbeat. When the heart contracts and blood is ejected from the left ventricle into the aorta, there is a change in the aortic pressure. Furthermore, this pressure fluctuation is propagated to the peripheral artery, and it propagates as strain to the body surface on the radial artery. The FBG sensor measures strain change due to pressure fluctuation. The signal of pulse wave measured by the FBG sensor is defined as a “pulse wave signal”.

In this measurement method, the FBG sensor is installed at the radial artery of the wrist, and a pulse wave signal is measured. The method is safe for the human body and does not involve the collection of blood. Since the blood glucose level is the glucose concentration in the blood, the blood flow will change owing to blood glucose level fluctuation and affect the pulse wave signal. If the FBG sensor can measure the pulse wave signal fluctuation, then the blood glucose level can be measured. Herein, the result of calculating the blood glucose level from the pulse wave signal measured using the FBG sensor and the prospect of non-invasive blood glucose level measurement by this method are described.

## 2. Experimental Design

### 2.1. FBG Sensor System

**Figure 1:**
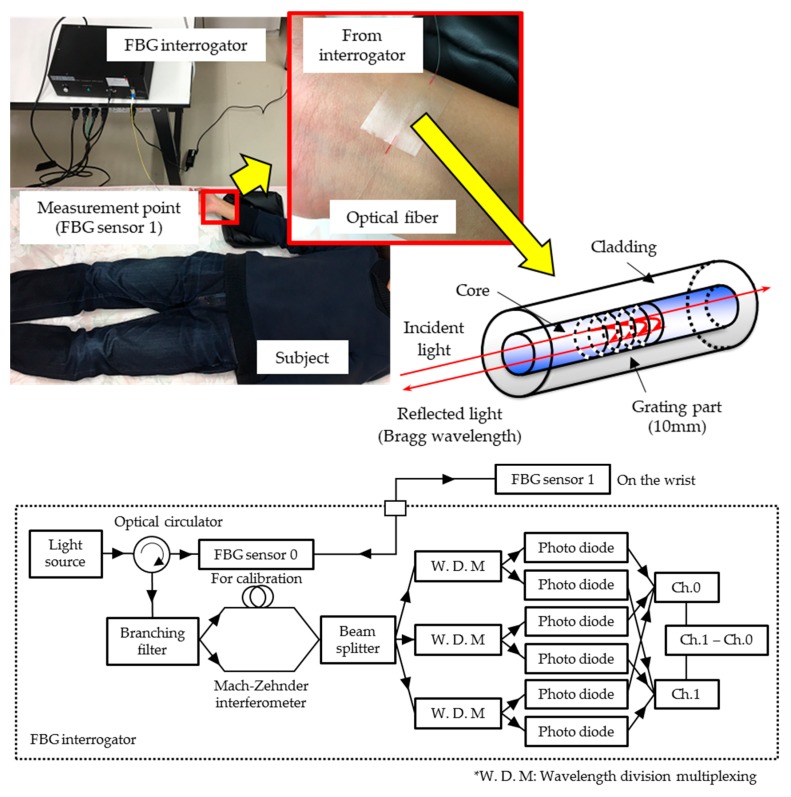
Photo and schematic diagram of the fiber Bragg grating (FBG) sensor system.

In this experiment, an FBG sensor system (PF25-S01: Nagano Keiki, Inc., Tokyo, Japan) was used [12]. This sensor system consists of an interrogator and an optical fiber. Figure 1 shows the photo and schematic diagram of the FBG sensor system. Broadband near-infrared (NIR) light with a wavelength range of 1525–1575 nm propagates through the optical fiber. Light reaches FBG sensor 1 through the optical circulator. The FBG sensor is a diffraction grating, in which the refractive index of the core of the optical fiber varies at equal intervals and has an optical filter function. In this diffraction grating, only a specific wavelength (Bragg wavelength) of NIR light from the light source is reflected, according to Equation (1), depending on the diffraction-grating spacing:

(1) $$\lambda_{\mathrm{Bragg}}={2n}_{\mathrm{eff}}\Lambda$$

where λ_Bragg_ is the Bragg wavelength, Λ is the diffraction-grating spacing, and n_eff_ is the refractive index inside the core. When distortion is applied to the sensor section, the diffraction-grating interval changes, because of which the Bragg wavelength also changes. This Bragg wavelength shift is measured by a Mach-Zehnder interferometer-type detection mechanism. The reflection light interferes in an interferometer, in which the optical path difference is set to 3.3 mm. A beam splitter splits the light into three components having phases that differ from each other by 2π/3 radians. The three phases are detected by wavelength division multiplexing. Three pairs of detectors detect the phase shifts of sensors 0 and 1, as shown in Figure 1. The signal for temperature correction of the measurement environment is measured by the FBG sensor 0. The phase resolution depends on the sampling frequency, which is 10 kHz. The FBG sensor measuring the pulse wave signal is shifted by 1.2 pm, with a strain of 1 με, and the measurement sensitivity is ±0.1 pm [13]. Using this system, the pulse wave signal was measured as a continuous signal showing a wavelength shift with respect to the time axis.

### 2.2. Pulse Wave Signal and Blood Glucose Level Measurement

Four subjects, who are all healthy males in their 20s, participated in the study. To measure the pulse wave signals, the FBG sensor was attached to the subject’s skin at the radial artery of the radial artery with a medical adhesive tape. In this measurement method, the strain of the artery that has propagated to the body surface is measured, so the calculated accuracy of the vital sign measurement is not affected by the color of the skin of the subject. Figure 1 shows the appearance of a typical pulse wave measurement in this study. The subject was in the supine position, and the wrist was kept as high as the heart. The measurement was performed for 20 s. 

**Figure 2:**
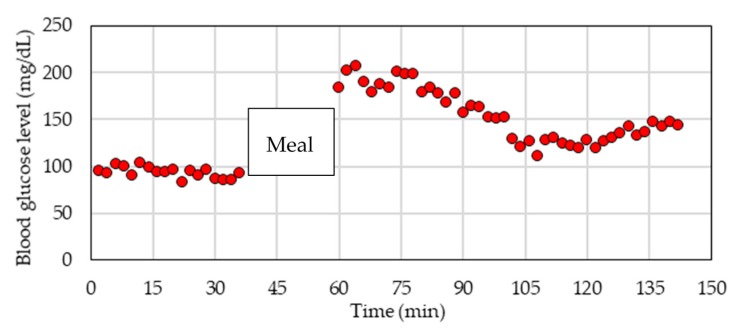
The time-series change in the blood glucose level (subject D).

Blood glucose was measured using an invasive blood glucose sensor, AntsenseIII (HORIBA Co., Ltd., Kyoto, Japan) or FreeStyle Precision Exceed H (Abbott Japan Co., Ltd., Osaka, Japan). This blood glucose level is used as the reference blood glucose when the pulse wave signal is measured. The relative uncertainty of the invasive blood glucose values in this reference method is 3.3–6.5%, when the glucose concentration is in the range of 90–220 mg/dL. In this experiment, the measurements were performed 20 times when the subject was in the fasting state, and they were performed another 40 times several hours after the subject had a meal. In the blood glucose level measurement experiments, blood glucose levels usually change with the oral glucose tolerance test (OGTT). In order to measure the blood glucose level, which is close to the usual life, we chose a method to change the blood glucose level by meal. Figure 2 shows the time-series change in the blood glucose level of subject D. All of the subjects gave informed consent before they participated in the study. The study was conducted in accordance with the Declaration of Helsinki, and the protocol was approved by the Ethics Committee of Shinshu University (No. 3202, Verification clinical trial with wearable vital sign measurement system.).

### 2.3. Blood Glucose Level Calculation Method

The pulse wave signal was filtered by a bandpass filter having a pass band of 0.5–5 Hz, and the signal was processed in the first differential. To calculate the blood glucose level from the pulse wave signal, the following four signal processing steps are necessary.

1. Division of the measured pulse wave signal at each peak by a 1-pulse pulse wave.
2. Averaging of a plurality of divided 1-pulse pulse wave signals.
3. Normalization of the vertical axis (wavelength shift) of the 1-pulse pulse wave.
4. Normalization of the horizontal axis (measurement time) of the 1-pulse pulse wave.

“1-pulse pulse wave” is a signal that is divided at the peak of a pulse wave and indicates a pulse wave signal in single beat of the heart. These processes are important for canceling fluctuations in the 1-pulse pulse wave signal measurement caused by pulse rate and respiration, as well as fluctuations due to the pressure of attachment the FBG sensor to the human body. For the pulse wave division, the peak due to the beat of the heart was selected.

In the normalization of the wavelength shift in one pulse wave signal, the first peak value is set to “1” , and the first valley value is set to “0” . The measurement time of the 1-pulse pulse wave was normalized using two methods. The first is normalization with the shortest measurement time (shortest-time-cut process). In this method, the measurement time is normalized by the shortest measurement time (approximately 0.7 s in this experiment) among the divided 1-pulse pulse wave signals. The signal at the back of the 1-pulse pulse wave is discarded, resulting in a reduction of the information in the pulse wave signal. For example, when the measurement time of the 1-pulse pulse wave signal is 0.8 s and the normalized time is 0.7 s, then the signal at 0.7–0.8 s is discarded. The second normalization method for the measurement time is to normalize all 1-pulse pulse wave signals to 1 s (1-s-normalization process). First, the measurement time is multiplied by an arbitrary constant, so that it is 1 s for the measured 1-pulse pulse wave. Next, a new point is created (linear interpolation) on a straight line connecting the nearest two points from 0.1 ms, and a similar calculation procedure is followed at the points of 0.2 ms, 0.3 ms, and so on to construct the 1-pulse pulse wave signal at 10,000 points within 1 s. By applying this normalization method for all 1-pulse pulse wave signals, all of the pulse wave signals are normalized to 1 s.

Using these signal-processed pulse wave signals and the reference blood glucose level, a calibration curve for calculating the blood glucose level is constructed by PLSR, which is a multi-variate analysis method. Since the reference blood glucose level (measured by the invasive blood glucose meter) has a measurement error, PLSR is suitable. Pulse wave signals were used as the explanatory variables, and the blood glucose levels, as measured by the invasive method, were used as the objective variables. Principle component analysis was performed for the pulse waves, and a feature vector called the PLS factor was extracted. In PLSR analysis, the objective variables (blood glucose levels) are expressed by a linear combination of the latent PLS factor of the explanatory variables (pulse waves). The residuals were used as the variables of the new model set for the next extraction step until the predicted residues of the objective values reach their minima [14]. The optimal numbers of PLS factors were tested statistically at a 5% significance level. The model set with the calculated optimum number of factors is used as the calibration curve for calculating the blood glucose level. In the validation of the calibration curve, pulse waves that were not used in the calibration were substituted to calculate the predicted blood glucose levels. The standard deviation of the error between this predicted blood glucose level and reference blood glucose level is the standard error of prediction (SEP).

In addition, error grid analysis (EGA) [15] was used for validating the calculation of blood glucose using this measurement method. EGA was used by Clark [16] to verify the clinical efficacy of blood glucose sensors. A scatter diagram with the reference blood glucose level on the horizontal axis and the blood glucose level calculated with the developed measurement method on the vertical axis is divided into five zones, labeled A–E [15]. EGA can verify the clinical efficacy in zones A and B, but not in zones D and E. We examine the proposed measurement method from the results of SEP and EGA.

## 3. Experimental Results and Discussion

### 3.1. Reference Blood Glucose Levels and Pulse Wave Signal of Each Subject

The pulse wave signal and reference blood glucose level were measured 60 times for each subject. The calibration curve for calculating the blood glucose level was constructed with 50 measurements (calibration data set), and the blood glucose level calculation was verified using the remaining 10 measurements (validation data set). Table 1 presents the calibration and validation data sets of the reference blood glucose level of each subject. When each subject had meals while being measured, blood glucose levels fluctuated from over 87 (subject C) to 139 (subject B) mg/dL.

**Figure 3:**
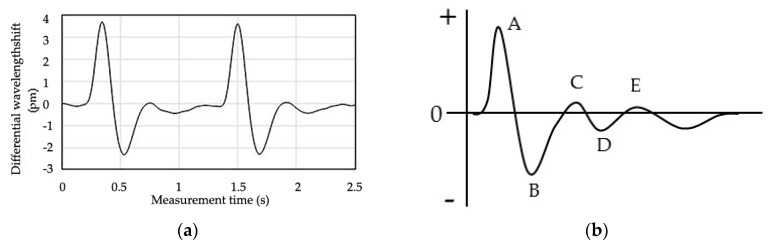
Measured pulse wave signal and basic acceleration plethysmogram. (**a**) Pulse wave signal measured with the FBG sensor; (**b**) Acceleration plethysmogram.

Figure 3 shows a pulse wave signal subjected to a first-derivative process in addition to a 0.5–5 Hz band pass filter, as well as a general acceleration plethysmogram. The acceleration plethysmo-gram has five peaks labeled A-E corresponding to the beating of the heart [17,18]. The measured pulse wave signal is very similar to the acceleration plethysmogram. Therefore, the signal measured by the FBG sensor system is a pulse wave signal including the information of blood flow from the heart.

**Figure 4:**
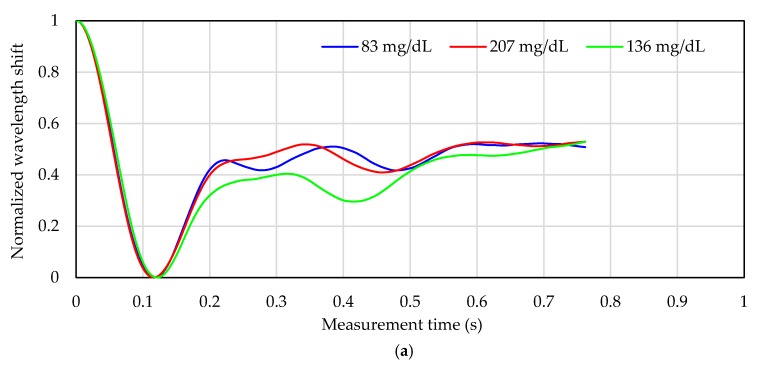

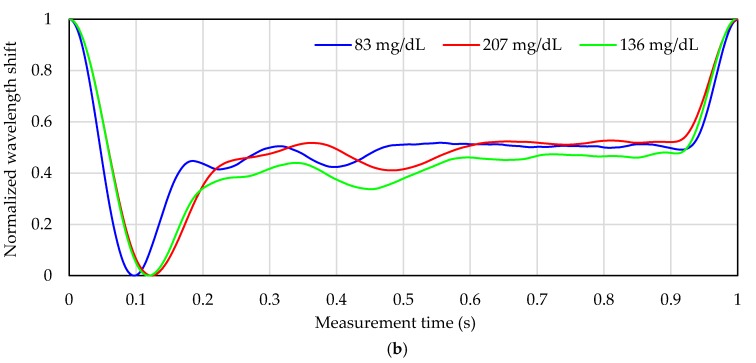
Pulse wave signal in each signal processing method. (**a**) Pulse wave signals in the shortest-time-cut process. (Blood glucose level, Min: 83 mg/dL, Max: 207 mg/dL, Ave.: 136 mg/dL); (**b**) Pulse wave signals in the 1-s-normalization process. (Blood glucose level, Min: 83 mg/dL, Max: 207 mg/dL, Ave.: 136 mg/dL).

Figure 4 shows the processing of the pulse wave signals for subject D with the two signal processing methods. The pulse wave signals are measured when the subject’s blood glucose concentration was maximum, minimum, and around the average value. In Figure 4a, the shortest time was 0.76 s; therefore, the pulse wave signal was cut at that time. Depending on the blood glucose level, the shape of the pulse wave signal is different at 0.2–0.6 s for each subject. In shortest-time-cut processing (Figure 4a), the measurement time points selected to be “0” for normalization are almost the same (around 0.12 s), but this measurement time point is different in the 1-s-normalization process. Therefore, in the 1-s-normalization process, a large difference appears in the slope of the peak at 0–0.1, 0.1–0.2, and 0.9–1 s. The shape of the acceleration plethysmogram depends on the blood flow and the hardness of the blood vessel. Therefore, since the glucose concentration in the blood flow varies depending on the blood glucose level, it is conceivable that the pulse wave shape is affected by the glucose concentration. The blood glucose level will be calculated by measuring the shape change of this pulse wave signal.

### 3.2. Blood Glucose Level Calculated by Calibration Curve

**Figure 5:**
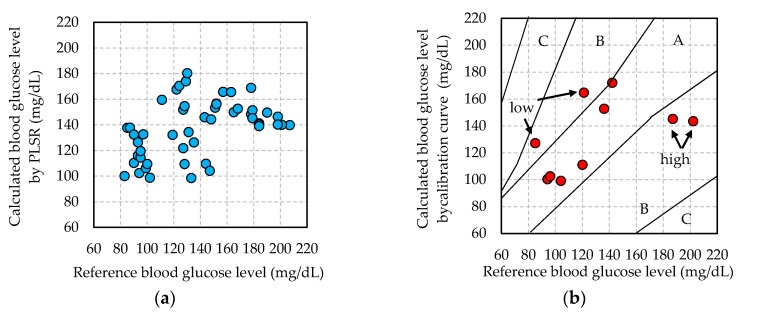

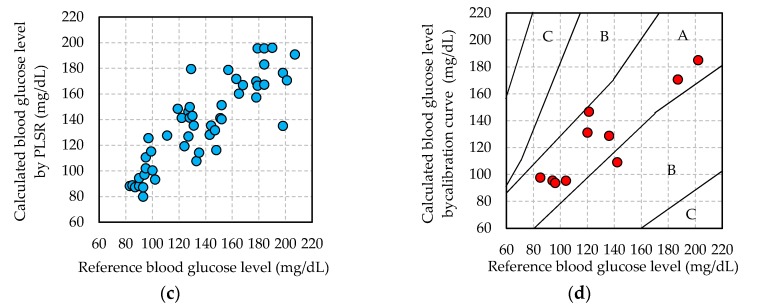
Calibration curve and validation results for calculated blood glucose level (Subject: D, shortest-time-cut and 1-s-normalization processing). (**a**) Sub.D-calibration curve in Shortest; (**b**) Sub.D-validation result in Shortest; (**c**) Sub.D-calibration curve in 1-s; (**d**) Sub.D-validation result in 1-s.

Since the blood flow changes with the glucose concentration, the blood glucose level is calculated from the calibration curve. The calibration curve is constructed from pulse wave signals that are subjected to the two signal processing methods, as described in Section 2.3. Figure 5 and Table 2 show the calibration curves, blood glucose level calculation, and EGA results for each subject in each signal process. The EGA results of all the subjects are plotted in the clinically effective zones A and B. However, SEP in the shortest-time-cut process was 26 mg/dL in subject D. Since the average blood glucose level in the validation data set of subject D is 129 mg/dL, SEP is approximately 21%. This result is remarkably poor.

On the other hand, in result of the 1-s-normalization process, the correlation coefficient of the calibration curve of three subjects exceeded 0.8, and in the EGA result, 80% of the data or more were plotted in zone A. Furthermore, SEP is 10–16 mg/dL, which is of the same level as the measurement error of commercially available invasive blood glucose measurement systems. The SEP of the 1-s-normalization process method was better than that of the shortest-time-cut processing, as indicated in Table 2. This SEP result is 9–12% of the average blood glucose value in the validation data set of Table 2. These results are very good for calculating blood glucose level.

The reason why the precision of blood glucose level calculation greatly differs by two signal processes is verified. In Figure 5b, the blood glucose levels calculated from the reference blood glucose levels 85, 121 mg/dL (“low” in the Figure 5b) and 187, 202 mg/dL (“high” in the Figure 5b) pulse wave signals were 127, 165 and 145, 143 mg/dL, respectively. These calculated blood glucose levels vary differ from the reference blood glucose level. In order to verify this cause, the calculated calibration curve is confirmed. In Figure 5a, the data on the reference blood glucose level of approximately 85 and 125 mg/dL are overestimated, and the data in approximately 180 and 200 mg/dL are underestimated. Therefore, the calibration curve that is constructed by this signal processing adversely affects the calculation of the blood glucose level. This phenomenon also appeared for subject B.

On the other hand, in Figure 5d of the validation result in the 1-s-normalization process, the same four reference blood glucose values are plotted around Y = X, and the blood glucose level is calculated with high accuracy. Accordingly, SEP values were also better with the 1-s-normalization process. The SEC value of the 1-s-normalization process for subject D is much better than those of the shortest-time-cut process. The calibration curve shown in Figure 5c is also plotted around the axis of Y = X. Therefore, in the 1-s-normalization process, reference blood glucose levels were correctly calculated. The data constituting these calibration curves are the same, and only the processing method of normalization of the horizontal axis (measurement time) of the 1-pulse pulse wave is different.

### 3.3. Adequacy of Non-Invasive Blood Glucose Measurement

In Section 3.2, it was shown that the calculation of blood glucose level is better with the 1-s-normalization process than with the shortest-time-cut process. In order to clarify the influence on the calibration curve by these processes, the loading vector of the PLS factor constructing the calibration curve is verified. A normalized pulse wave signal for a blood glucose level close to the highest, lowest, and average values for subject D processed by each normalization method is shown in Figure 4. The normalized wavelength shift of the pulse wave signal in the shortest-time-cut process is 0 at approximately 0.12 s for all of the blood glucose levels, and the pulse wave signal after approximately 0.75 s has been deleted. On the other hand, in the 1-s-normalization process, the measurement time of the pulse wave signal at which the wavelength shift is 0 varies among different blood glucose levels. 

**Figure 6:**
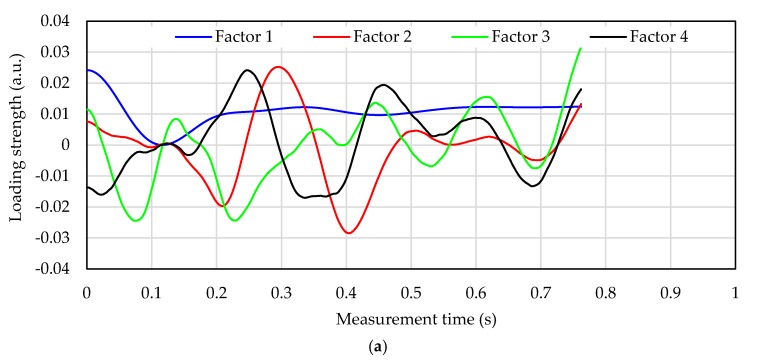

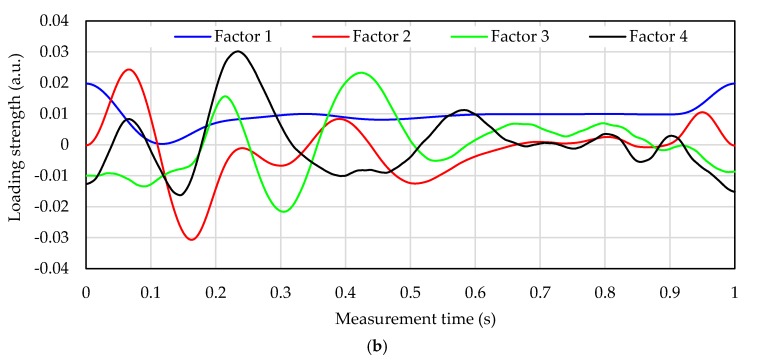
Loading vector of calibration curve in subject D. (**a**) Loading vector of calibration curve in shortest-time-cut process; (**b**) Loading vector of calibration curve in the 1-s-normalization process.

Figure 6 shows the loading vector of each factor that is used for constructing the calibration curve in each processing method for subject D. The loading vector indicates the dependence of each factor on the calibration curve. The greater the absolute value on the vertical axis is, the more significantly the wavelength shift at that time depends on the blood glucose level calculation. In Figure 6, the loading vector at Factor 1 in each processing method is similar to the pulse wave signal. In the loading vector of factor 2 (Figure 6b red line) of the calibration curve in “1-s-normalization process” with high calculation accuracy, the absolute values are 0.07 s and 0.16 s on the positive and negative side, respectively. The numerical value on the vertical axis at Factor 1 is 0 at 0.12 s. Therefore, the loading vector of factor 2 affects the change in inclination around peak B in Figure 4b. These inclinations are affected by the time-axis direction (horizontal-axis direction), since peak A in Figure 3b is normalized to “1” and peak B is normalized to “0.” Furthermore, in the loading of the 1s-normalization process in Figure 6b, Factor 1 has a large peak after 0.92 s, and Factor 2 has a large peak at 0.95 s. These are the rising parts of peak A in Figure 3b. Therefore, normalized calibration curves that capture the features of inclinations around peaks A and B is calculated the calculation of blood glucose level in high accuracy.

On the other hand, in the loading vector of factor 2 (Figure 6a red line) of the calibration curve in “shortest-time-cut process s” with low calculation accuracy, the absolute value is 0.3 s on the positive side and 0.4 s on the negative side. It shows almost 0 at 0.07 and 0.16 s. At these measurement times, the absolute value of loading of factor 3 (Figure 6a green line) is large, however this value is smaller than the absolute value of factor 2 in “1-s-normalization process”. Therefore, the change in inclination around the peak B in Figure 3b is not shown in each factor of the calibration curve in “shortest-time-cut process”. In addition, the absolute values of factors 2, 3, and 4 after 0.7 s are large, and this information has a big influence. However, in Figure 4a, since there are no characteristic peaks after 0.7 s, information unrelated to the pulse wave signal is indicated in the factor. Since the rising part of peak A in Figure 3b after 0.92 s has been deleted by “shortest-time-cut process”, the influence of the pulse wave signal in this part is not included in each factor.

From the above, the calculation of the blood glucose level from the pulse wave signal is greatly affected by signal processing on the “Measurement time” axis, which is a feature of the “1-s-normalization process”. In the 1-s-normalization process, this influence of the time-axis direction is well captured. The calculation of the blood glucose level is significantly influenced by the inclination of the pulse wave signal around the peak A and B in Figure 3b. In other words, the blood glucose level is not exactly the magnitude of the pulse; rather, it is significantly dependent on the blood flow in the time-axis direction.

The causes of changes in the blood flow due to the blood glucose level may be as follows.

- More glucose was contained in blood after a change in the blood glucose level; consequently, the blood flow changed because of a change in blood viscosity.
- Since glucose is sent into the body, the blood vessels expanded at the time of hyperglycemia, and the blood flow changed.

Medical verification to confirm these causes is a future task.

## 4. Conclusions

This paper reported a revolutionary method of non-invasive blood glucose measurement using an FBG sensor system. Pulse wave signals were measured for four subjects, and blood glucose levels were calculated using two signal processing methods. Consequently, we found that the blood glucose level that was calculated with the shortest-time-cut process had poor measurement accuracy above 200 mg/dL. The blood glucose level calculated with the 1-s-normalization process had good measurement accuracy overall. Moreover, the acquisition of the slopes of peaks A and B of the pulse wave signal from the loading vector of the calibration curve in each signal processing method improved the accuracy of calculation of the blood glucose level. Lastly, we found that to calculate the blood glucose level from the pulse wave signal with high accuracy, the blood flow should be considered.

Our results indicate that the blood glucose level can be reliably calculated from the pulse wave signal measured by the FBG sensor. However, it is necessary to medically verify the relationship between the blood glucose level and blood flow. When this relationship becomes clear, the calculation of blood glucose level from the pulse wave signal would be theoretically validated.

To perform this verification, it is necessary to investigate the blood flow while the blood glucose level is changing. However, it is not possible to measure the blood flow by performing incisions on the subject. Therefore, we plan to use ultrasonic tomographic imaging equipment [19,20,21], which can image the inside of the body from the outside, to measure the blood flow. In this experiment, this device will be placed at the same location as the FBG sensor: the radial artery of the subject. The subject's blood glucose level will be intentionally changed, and simultaneous measurements will be performed using the FBG sensor and the ultrasonic tomographic imaging device. Then, the blood flow and the diameter of the blood vessel will be measured using the ultrasonic tomographic imaging device. The relationship between the measurements of the ultrasonic tomographic imaging device and the shape of the pulse wave signal detected by the FBG sensor will be investigated for each blood glucose level.

We have already reported that the pulse rate, respiration rate, and blood pressure can be calculated simultaneously and continuously from the pulse wave signal that is measured using an FBG sensor system [22,23,24]. If non-invasive blood glucose measurement is also included the abovementioned list, an FBG sensor system can be used as a convenient multi-vital-sign sensor. For diabetic patients, we aim for real-world implementation as soon as possible.

## Figures and Tables

**Table 1:** Reference blood glucose data set.

Subject (Gender)	Number of Measurements	Blood Glucose Level (mg/dL)		
		Maximum	Minimum	Average
Calibration Data Set				
**A (male)**	50	178	80	119
**B (male)**	50	232	93	143
**C (male)**	50	176	89	127
**D (male)**	50	207	83	138
Validation Data Set				
**A (male)**	10	153	82	113
**B (male)**	10	188	97	138
**C (** **male** **)**	10	164	89	115
**D (male)**	10	202	85	129

**Table 2:** Calibration curve and validation results for each subject.

Subject		A		B		C		D	
Processing Method		Shortest	1-s	Shortest	1-s	Shortest	1-s	Shortest	1-s
**Calibration result**	SEC (mg/dL)	17	15	34	21	15	14	33	19
	r	0.67	0.77	0.58	0.86	0.84	0.87	0.44	0.86
	factors	4	4	4	4	4	4	4	4
**Validation result**	SEP (mg/dL)	20	10	23	16	7	12	26	14
	A-zone (%)	60	80	80	80	100	100	50	90
	B-zone (%)	40	20	20	20	0	10	50	10
